# A Crossover Randomized Controlled Trial of Priming Interventions to Increase Hand Hygiene at Ward Entrances

**DOI:** 10.3389/fpubh.2021.781359

**Published:** 2022-01-17

**Authors:** Kelly Ann Schmidtke, Navneet Aujla, Tom Marshall, Abid Hussain, Gerard P. Hodgkinson, Kristopher L. Arheart, David J. Birnbach, Laura Kudrna, Ivo Vlaev

**Affiliations:** ^1^Warwick Medical School, The University of Warwick, Coventry, United Kingdom; ^2^Faculty of Medical Sciences, Newcastle University, Newcastle Upon Tyne, United Kingdom; ^3^Primary Care Clinical Sciences Institute of Applied Health Research College of Medical and Dental Sciences, University of Birmingham, Birmingham, United Kingdom; ^4^Public Health England, Public Health Laboratory, Birmingham Heartlands Hospital, Birmingham, United Kingdom; ^5^Manchester Institute of Innovation Research, Alliance Manchester Business School, The University of Manchester, Manchester, United Kingdom; ^6^Division of Biostatistics, Department of Public Health Sciences, University of Miami Leonard M. Miller School of Medicine, Miami, FL, United States; ^7^Department of Anesthesiology, University of Miami Leonard M. Miller School of Medicine, Miami, FL, United States; ^8^Institute of Applied Health, University of Birmingham, Birmingham, United Kingdom; ^9^Behavioral Science Group, Warwick Business School, The University of Warwick, Coventry, United Kingdom

**Keywords:** hand hygiene, priming, theoretical domains framework (TDF), quality improvement, behavior change

## Abstract

**Background:**

Research conducted in the United States suggests that two primes (citrus smells and pictures of a person's eyes) can increase hand gel dispenser use on the day they are introduced in hospital. The current study, conducted at a hospital in the United Kingdom, evaluated the effectiveness of these primes, both in isolation and in combination, at the entry way to four separate wards, over a longer duration than the previous work.

**Methods:**

A crossover randomized controlled trial was conducted. Four wards were allocated for 6 weeks of observation to each of four conditions, including “control,” “olfactory,” “visual,” or “both” (i.e., “olfactory” and “visual” combined). It was hypothesized that hand hygiene compliance would be greater in all priming conditions relative to the control condition. The primary outcome was whether people used the gel dispenser when they entered the wards. After the trial, a follow up survey of staff at the same hospital assessed the barriers to, and facilitators of, hand hygiene compliance. The trial data were analyzed using regression techniques and the survey data were analyzed using descriptive statistics.

**Results:**

The total number of individuals observed in the trial was 9,811 (female = 61%), with similar numbers across conditions, including “control” *N* = 2,582, “olfactory” *N* = 2,700, “visual” *N* = 2,488, and “both” *N* = 2,141. None of the priming conditions consistently increased hand hygiene. The lowest percentage compliance was observed in the “both” condition (7.8%), and the highest was observed in the “visual” condition (12.7%). The survey was completed by 97 staff (female = 81%). “Environmental resources” and “social influences” were the greatest barriers to staff cleaning their hands.

**Conclusions:**

Taken together, the current findings suggest that the olfactory and visual priming interventions investigated do not influence hand hygiene consistently. To increase the likelihood of such interventions succeeding, future research should focus on prospectively determined mechanisms of action.

## Introduction

The National Health Service in the United Kingdom spends more than £1 billion per annum on healthcare-associated infections (1). Increasing hand hygiene could reduce this cost and, in so doing, reduce considerably morbidity and mortality (2), an issue which has gained increased salience in the context of the ongoing coronavirus pandemic (3). To promote hand hygiene, hospitals ask staff and visitors to use antiseptic gel and soap dispensers. Many people do not comply with this request (4, 5). Lack of compliance could stem from more reflective (i.e., deliberative) or more automatic, reflexive (i.e., non-deliberative) psychological processes, as posited by dual-process theories (6).

To improve hand hygiene in hospital settings, the World Health Organization's 2009 multimodal strategy recommends simultaneously attending to five component issues that might engage both the reflective and automatic processes, including: system change, training and education, observation and feedback, hospital safety climate, and reminders (7). These recommendations remain the same during the pandemic. Broadly, most hospital infection prevention and control policies already address many of these components. For example, a 2018 meta-review of systematic reviews located 16 reviews that included studies involving training and education interventions and 15 involving reminder interventions (8). Training and education interventions largely attempt to engage people's reflective processes, whereas reminder interventions mainly focus on engaging their automatic processes. In this research, we focus on the latter approach, predicated on the assumption that most people (staff, patients, and hospital visitors) already reflectively know how and why to clean their hands: they just need a little nudge to actualize their good intentions.

Automatic mechanisms of behavior change (also known as behavioral determinants), as well as their triggers or techniques (also known as “nudges”) are well-documented in the behavioral economics literature (9). The behavior change techniques employed in the present trial are commonly referred to as psychological priming techniques (10). Psychological priming is a process by which exposure to a certain cue (e.g., particular smells or images, known as primes), activates mental concepts that alters behavior, without the person being aware of the impact of the cue on their behavior, even though they may be aware of the cue itself (11). Psychological primes are typically physical cues that make particular behaviors more accessible in memory, and, in so doing, increase the likelihood that those behaviors will occur (12, 13).

Our interest in using primes to increase hand hygiene compliance was stimulated by a study that used an olfactory prime (a citrus smell) to encourage people to keep their surrounding environment clean (14), and a second study that used visual primes (pictures of people's eyes) to encourage prosocial behaviors (15). These studies highlighted key mechanisms for promoting behavior change that might generalize to hand hygiene in hospital settings. In the first of these studies, medical interns and students were asked to examine an actor complaining of heart palpitations in either a citrus smelling room or in an unmodified room (16). Participants in the citrus smelling room were more likely to clean their hands before touching the actor. In the second study, staff and visitors were more likely to use a gel dispenser when they entered a surgical unit if a citrus-smell or a picture of a person's eyes were placed above that gel dispenser than when neither prime was present (17). Plausibly, citrus smells activate concepts in people's memory related to cleaning products, which in turn then trigger hygienic behavior, whereas pictures of a person's eyes remind people of social norms that encourage pro-social behavior. Both studies were conducted in the United States. The present research examines whether the benefits of these olfactory and visual priming interventions generalize to a hospital in the United Kingdom.

There is general agreement that subtle primes can influence behavior, but the reliability of such primes is debated (18). While the primary focus of the present research is of a practical nature (i.e., to assess the suitability of olfactory and visual prime interventions for promoting gel dispenser use in hospital wards), it also contributes to the growing basic literature on primes in the following ways. First, the trial assesses whether primes found to be effective over brief durations (minutes or hours) maintain their effectiveness over longer durations (6-weeks); that is, it examines the longevity of their effects. Secondly, the trial assesses whether olfactory and visual primes found to be effective in isolation have greater or lesser benefits when combined. Thirdly, the present research assesses whether primes found to be successful in promoting the use of one dispenser type (i.e., gel dispensers) generalize to another dispenser type (i.e., soap dispensers). Finally, to the extent that our results confirm that the primes under investigation are effective, the present research would extend the boundary conditions pertaining to those previously reported in the United States and demonstrate that the findings generalize to the United Kingdom.

The primary focus of the present research is to assess the suitability of olfactory and visual prime interventions for promoting hand hygiene behavior in hospital wards in the United Kingdom. Our findings suggest they do not. Our three objectives and related predictions are briefly provided below.

Objective 1 – Gel/soap dispenser use: Record people's use of the gel and soap dispensers upon entering the wards when either, both, and neither prime is present. We predict that the proportion of people who use the dispensers will be greater when the primes are present than when they are not, and that the combined priming condition will exhibit the highest rates of hand hygiene compliance.

Objective 2 – Gel/soap material use: Record the amount of material used from the dispensers each week when either, both, and neither prime is present. These findings should corroborate the findings pertaining to Objective 1.

Objective 3 – Individual differences: Record the role at hospital and gender of people entering the wards to see if different types of people respond differentially to the primes.

## Methods

The research methods pertaining to the present trial are fully described in the open-access protocol [http://dx.doi.org/10.1136/bmjopen-2017-017108, (19)]. The trial was ethically approved by the Health Research Authority (16/SC/0554). Data collection began on 10 March 2017 and ended on 15 September 2017. The trial was registered at https://doi.org/10.1186/ISRCTN15397624. The application to register the trial protocol (with an analysis plan) was submitted on the 28 February 2017, before the 1st day of data collection, but the registration was delayed due to payment issues until the 31 March 2017.

### Design and Setting

Four wards in a single hospital were assigned to all four conditions at different times in a crossover randomized controlled trial. The four wards specialized, respectively, in renal medicine (ward A), gastrointestinal surgery (ward B), hematology/oncology (ward C), and admission (ward D). These wards were selected by the hospital's Director of Infection Prevention and Control due to their history with healthcare associated infections. Minor deviations were made from the published protocol. First, to ensure sufficient observations, the observation period was increased from 2 to 4 h in the third trial phase (see the “Sample size” section), this was approved as an amendment to the stated ethical approval. Second, given the findings pertaining to the primary outcome, two of the objectives set out in the protocol (19) were not explored further, as they were no longer relevant, namely: (1) comparing the number of infections and (2) calculating the cost-effectiveness of the interventions.

### Participants

The handwashing behaviors of all people walking onto the wards during the observation sessions were recorded. The fact that a trial was being conducted to improve hand hygiene was communicated in the Trust's newsletter, but participation in the trial was incidental as researchers recorded the behavior of anyone who walked onto the ward during observation session.

### Sample Size

The sample size calculations for the primary analysis (28 per observation session) were based on a projected “control” group hand hygiene rate of 15–20% and a clinically significant increase of 15%, to give us 93% power for a one-tailed test, and 87% power for a two-tailed test. The 15% increase was informed by the effects found in the previous study (16). Initially, observations were scheduled for 2 h per ward each week, i.e., 1 h on Monday and 1 h on Wednesday mornings. The initial week's data collection suggested that these sessions might yield an insufficient number of observations, and so an amendment was approved, starting in phase 3. The amendment increased observations to 4 h per ward each week, by adding one extra hour per ward on Monday afternoons and one extra hour per ward on Wednesday afternoons.

### Randomization

The wards were observed under each of four conditions: “control,” “olfactory,” “visual,” or “both.” Each condition was active for 6-weeks, followed by a 1-week washout period when no primes were active. The order in which each condition was scheduled to be active on each ward was randomly assigned according to a Latin square design (20), allocated by the first author of this paper. This design and description of these trial phases are shown in Table 1. While hospital staff and visitors were not told when the interventions were active, due to the nature of the interventions, blinding and allocation concealment was not feasible.

**Table 1:** Observation schedule.

	**1st Phase**		**2nd Phase**		**3rd Phase**		**4th Phase**
	**Observe weeks**	**Washout week**	**Observe weeks**	**Washout week**	**Observe weeks**	**Washout week**	**Observe weeks**
Ward entrance	1–6	7	8–13	14	15–20	21	22–27
A	Both	Control	Visual	Control	Control	Control	Olfactory
B	Control	Control	Both	Control	Olfactory	Control	Visual
C	Olfactory	Control	Control	Control	Visual	Control	Both
D	Visual	Control	Olfactory	Control	Both	Control	Control

### Interventions

The olfactory prime was a citrus-smell dispensed from ScentDirect diffusers (21). The wards were spaced sufficiently apart such that the olfactory prime was not perceptible across them. The visual prime was a laminated picture of the eye region of a person's face posted above the observed gel and soap dispensers.

### Outcomes

The primary outcome was the percentage of occasions on which people entering the wards used a gel dispenser. This is the same primary outcome measure used in the previous studies (8, 9). The secondary outcomes were the percentage of occasions on which people entering the wards used a soap dispenser, and the amount of gel material and soap material used each week.

### Data Collection

#### Dispenser Use

Only one researcher observed each site at any given time. Researchers stood in full view of the dispensers without disturbing typical practice. A counter-balanced schedule was used that rotated the wards and hours of observation. On paper templates, the researchers recorded each participants' apparent gender (female, male), role at hospital (doctor, nurse, other staff, visitor), gel-dispenser-use (yes, no), and soap-dispenser-use (yes, no). The data were transferred to Excel files on the same day they were collected. A third researcher reviewed the paper and Excel files for five randomly selected days and found that 98.4% of the data were transferred accurately. Additional observation sessions were conducted to assess inter-rater reliability. Both researchers simultaneously recorded hand hygiene activity for 15 min on each ward. During these additional observation periods, they agreed that 36 people entered the ward. The observed Kapa statistics for gel use were good (*K* = 0.79; 95% CI, 0.38–1.00), were perfect for soap use and gender (*K* = 1.00; 95% CI, 1.00–1.00), and were nearly perfect for role (*K* = 0.93; 95% CI, 0.82–1.00).

#### Cleansing Material Use

Data regarding the amount of cleansing materials used were collected by weighing gel and soap containers each Friday between 15:00 and 17:00. In total, 208 gel and 112 soap measurements were taken.

### Statistical Analyses

Statistical analyses were carried out using Statistical Analysis System 9.4 (SAS Institute Inc., Cary NC). The descriptive statistics provide the frequency and percentage of occasions that people used the gel and soap dispensers on each ward, broken down by condition, gender, and role.

The first set of analyses involve use of the gel dispenser and the soap dispenser to achieve objectives 1 (about gel and soap dispenser use) and 3 (about individual differences at the gel dispenser only). As gel dispenser use was the primary outcome, the primary analysis is the model for gel dispenser use. As per the protocol (19), a two-factor omnibus model was computed, which examined the main effects of phase (1, 2, 3, 4) and ward (A, B, C, D), together with the interaction effect of phase × ward for gel dispenser use and then for soap dispenser use. For gel dispenser use, a significant carryover effect was found, and, as per the protocol, phase 1 data were analyzed separately. Reflecting on this original analysis plan, the research team noted that the use of phase 1 only data confounds ward and intervention. This would not be a problem if the hand hygiene rates were similar across wards, but that is not the case here. Thus, we then decided to conduct additional *post-hoc* sensitivity analyses, to assess whether the results from phase 1 held when using data from all phases.

The *post-hoc* sensitivity analyses were generalized linear mixed models for binomial outcomes. A random intercepts model was fitted with weeks nested within phases as the error term. The correlated data structure was represented by a heterogeneous compound symmetric covariance matrix for the best possible model fit. The outcomes were gel dispenser use (yes/no) for the first analysis, and then soap dispenser use (yes/no) for the second. The predictors included condition (“control,” “olfactory,” “visual,” “both”), ward (A, B, C, D), and the interaction between condition and ward. For gel use we also assessed the influence of the individual difference variables for gender (female, male), role (doctor, nurse, other staff, visitor), and the three-way interaction of condition × gender × role.

The second set of analyses involved use of the gel material, and soap material use to achieve objective 2 (compare material use across conditions).

For all models, four planned comparisons were made between each of the single prime conditions and the “control” condition, and between each of the single prime conditions and the “both” conditions. The alpha level was set to 0.05 to determine statistical significance, adjusted for multiple comparisons using the Tukey-Kramer method.

## Results

### Descriptive Statistics

**Figure 1:**
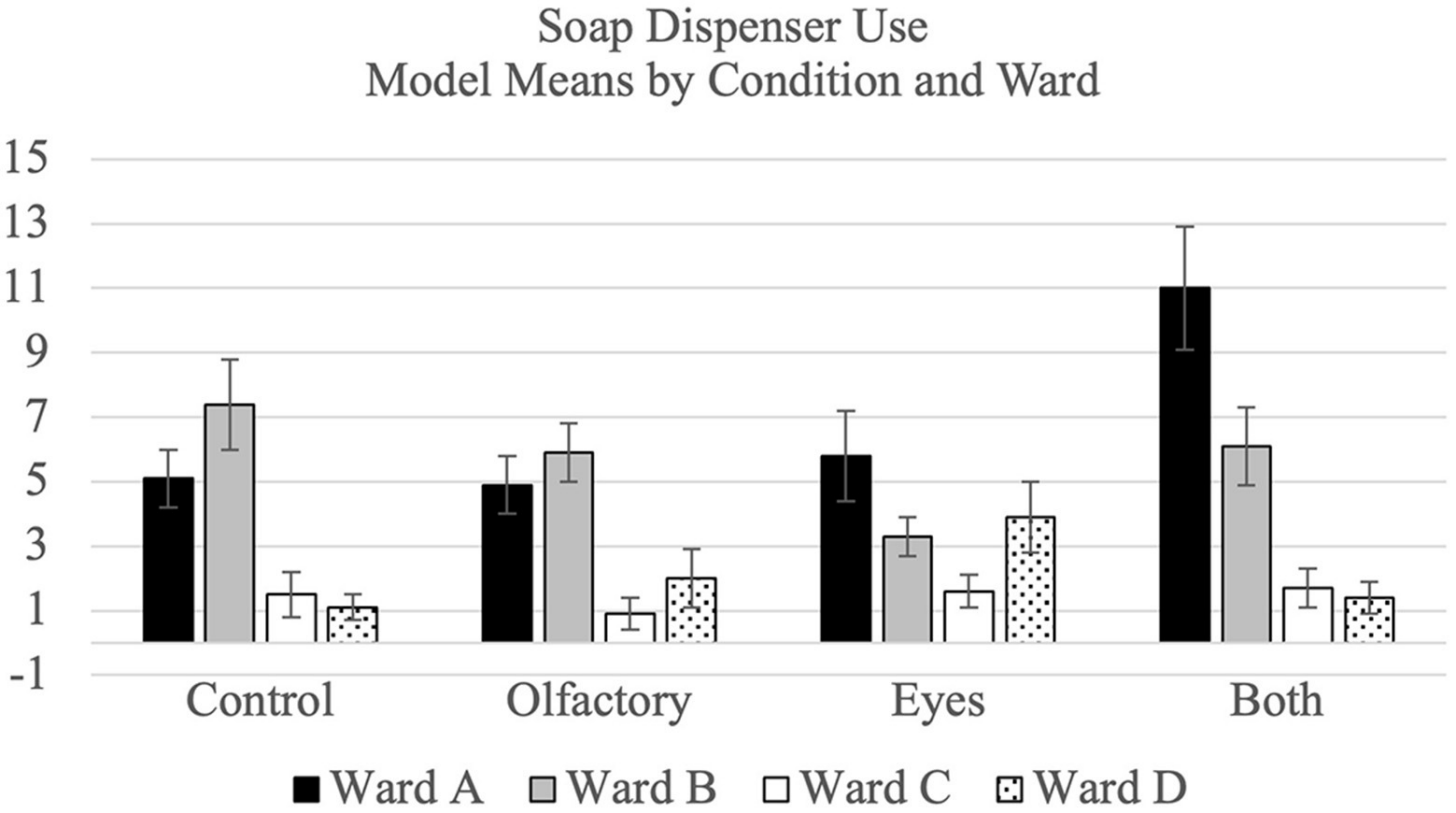
Consort flow chart.

The descriptive statistics pertaining to gel and soap dispenser use, broken down by ward, treatment condition, gender, and role at hospital are summarized in Table 2. The consort diagram is provided in Figure 1. The total number of observations recorded at the entrances to the wards was 9,811 (female = 5,963, 60.8%). A higher number of observations were recorded on wards A and B than C and D, and gel and soap dispenser use were higher on wards A and B than C and D. Gel dispenser use was highest during the “visual” condition and lowest in the “both” condition, while soap dispenser use was similar across all conditions.

**Table 2:** Descriptive statistics pertaining to the use of hand cleansing materials, broken down by ward, treatment condition, gender, and role at hospital.

	**Number of observations** **(percentage of total)**		**Percentage Gel-Dispenser-Use**	**Percentage Soap-Dispenser-Use**
Total		9,811 (100.0)		
Ward	A	2,653 (27.0)	15.7	6.0
	B	3,377 (34.4)	12.8	5.1
	C	1,910 (19.5)	9.1	1.5
	D	1,871 (19.1)	3.0	1.9
Treatment condition	Control	2,482 (25.3)	11.9	3.9
	Olfactory	2,700 (27.5)	11.0	4.5
	Visual	2,488 (25.4)	12.7	3.3
	Both	2,141 (21.8)	7.8	4.3
Gender^a^	Female	5,963 (60.8)	11.0	5.1
	Male	3,847 (39.2)	11.0	2.3
Role at hospital^b^	Doctor	1,122 (11.4)	11.9	2.5
	Nurse	1,847 (18.8)	9.4	4.5
	Other Staff	3,619 (36.9)	9.4	6.4
	Visitors	3,198 (32.6)	13.4	1.7

a *Gender observation was missing*.

b *Role observations were missing*.

Gel dispenser use was descriptively similar for males and females, while soap dispenser use was higher for females than males. Regarding role, most observations were recorded as other staff, followed by visitors, nurses, and doctors. Gel dispenser use was highest for visitors, followed by doctors, and then equally by nurses and other staff. In contrast, a nearly reverse trend for soap dispenser use appeared, with the highest use for other staff, followed by nurses, doctors, and visitors.

### Models

#### Dispenser Use—Objective 1

**Figure 2:**
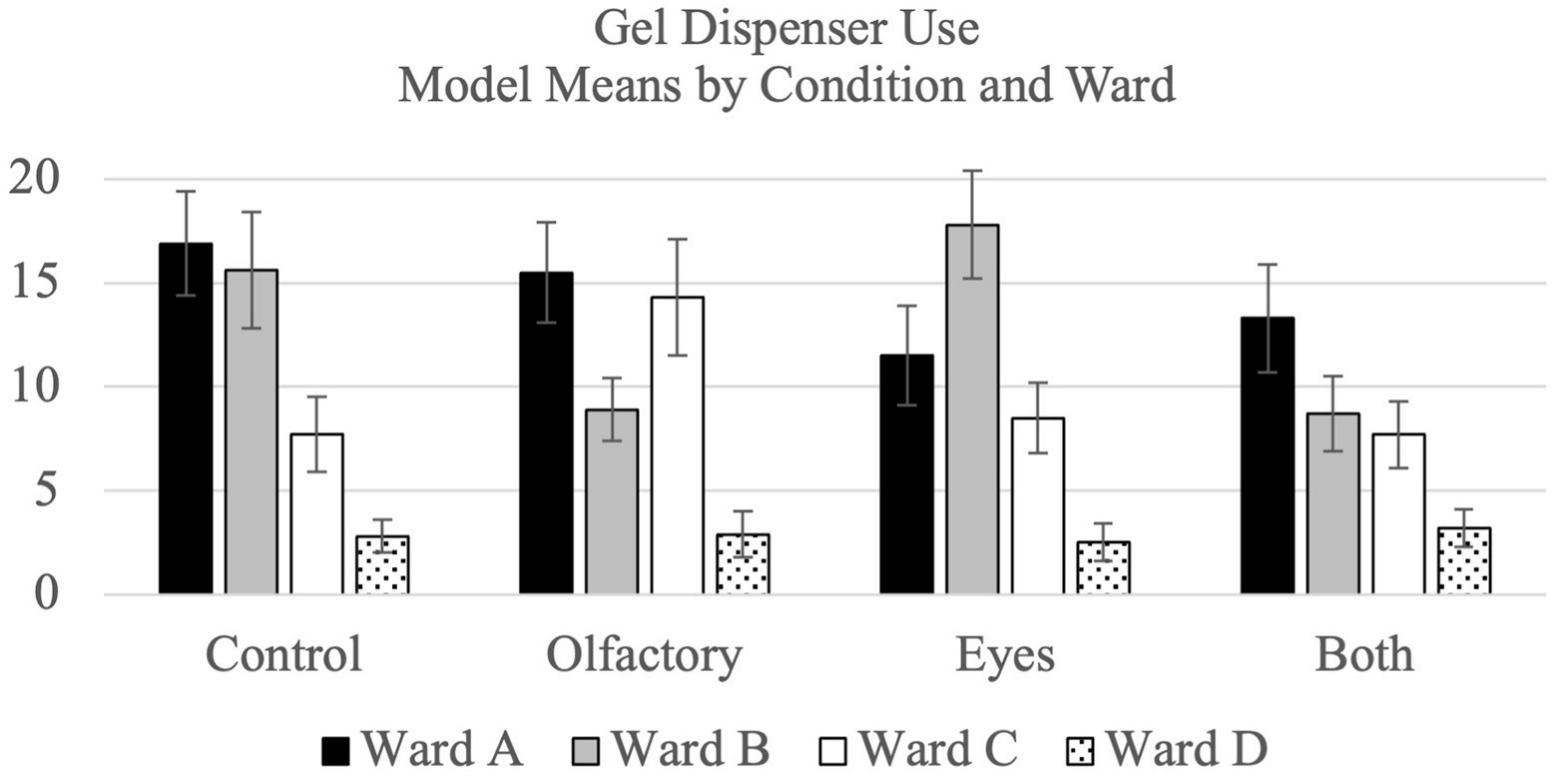
Modal mean percentage for hygiene compliance broken down by treatment condition and ward for gel dispenser use and soap dispenser use separately.

The model means and standard errors pertaining to for hand hygiene by condition and ward for gel and soap dispenser use are shown visually in Figure 2. As per the protocol, a test for carryover effects in the crossover design was conducted. The result was significant (*p* < 0.001), and, accordingly, only phase one data were analyzed. The results showed that the olfactory and visual priming conditions were *less* effective than the control condition (all *p*s < 0.05), and that they were each less effective than the both condition, too (all *p*s < 0.05). These results are reported to remain consistent with the protocol and are further summarized in Supplemental Materials 1. Limitations of the protocol analysis (that phase 1 confounds ward and condition) led us to conduct *post-hoc* sensitivity analyses.

In the *post-hoc* sensitivity analyses using all phases, for gel use, there was no significant difference between each single priming conditions and the control condition (all *p*s > 0.05), and no difference between each single priming condition and the “both” condition (all *p*s > 0.05). In the *post-hoc* sensitivity analyses, for soap use, no consistent differences emerged across the wards. On ward A, soap use was higher during the both condition than during the control condition (*p* < 0.01) and smell condition (*p* < 0.01). On ward B, the soap use was higher during the control condition than the eyes condition.

#### Material Use—Objective 2

The model means and standard errors pertaining to for material use by condition and ward for soap and gel dispensers are provided in Table 3. For gel use, when each single priming condition was compared to the control condition, no significant differences emerged (all *p*s > 0.5). When each single priming condition was compared to the both condition, again, no significant differences were found (all *p*s > 0.05).

**Table 3:** Model means and standard errors (SE) for gel and soap material-use in grams, broken down by treatment condition and ward.

**Ward**	**Condition**	**Gel**	**Soap**
		**Mean (SE)**	**Mean (SE)**
A	Both	636 (82)	483 (48)
	Control	781 (73)	422 (48)
	Eyes	926 (82)	432 (48)
	Smell	570 (73)	478 (48)
B	Both	210 (73)	295 (48)
	Control	380 (94)	337 (48)
	Eyes	425 (73)	313 (48)
	Smell	207 (73)	304 (48)
C	Both	491 (73)	102 (48)
	Control	642 (73)	100 (48)
	Eyes	485 (73)	96 (48)
	Smell	662 (73)	62 (48)
D	Both	127 (73)	230 (48)
	Control	170 (73)	209 (48)
	Eyes	112 (73)	292 (48)
	Smell	172 (82)	265 (48)

#### Individual Differences—Objective 3

The model means and standard errors gel dispenser use are further broken down by gender, in Table 4 and by role at hospital in Table 5. The two-way interactions of gender × condition and role × condition were not significant (all *p*s > 0.05).

**Table 4:** Model means and standard errors (SE) pertaining to gel-dispenser-use and soap-dispenser-use, broken down by treatment condition, ward, and gender.

**Gel-Dispenser-Use**		**Female**	**Male**
**Ward**	**Condition**	**Mean (SE)**	**Mean (SE)**
A	Both	18.1 (4.5)	14.0 (4.4)
	Control	16.6 (3.6)	16.4 (3.7)
	Eyes	8.6 (2.8)	14.1 (4.2)
	Smell	15.5 (3.5)	11.7 (3.0)
B	Both	7.0 (2.2)	8.4 (2.9)
	Control	15.8 (3.9)	15.8 (4.6)
	Eyes	13.8 (3.1)	21.5 (4.5)
	Smell	9.5 (2.3)	7.6 (2.2)
C	Both	7.7 (2.4)	11.4 (3.5)
	Control	9.3 (3.3)	8.1 (3.2)
	Eyes	8.4 (2.6)	5.3 (1.9)
	Smell	13.4 (4.2)	12.7 (4.4)
D	Both	2.7 (1.1)	4.3 (1.6)
	Control	4.2 (1.5)	1.0 (0.7)
	Eyes	4.1 (1.8)	1.7 (1.3)
	Smell	3.2 (1.7)	1.9 (1.2)

**Table 5:** Model Means and standard errors pertaining to gel-dispenser-use and soap-dispenser-use, broken down by treatment condition and role in hospital.

**Gel-Dispenser-Use**		**Doctor**	**Nurse**	**Other staff**	**Visitor**
**Ward**	**Condition**	**Mean (SE)**	**Mean (SE)**	**Mean (SE)**	**Mean (SE)**
A	Both	26.0 (7.6)	26.2 (8.0)	7.1 (2.5)	11.9 (4.3)
	Control	23.0 (6.1)	8.5 (2.7)	16.5 (3.7)	21.9 (4.5)
	Eyes	14.1 (6.1)	6.0 (3.0)	10.5 (3.5)	16.2 (5.2)
	Smell	11.1 (4.0)	9.3 (2.9)	15.1 (3.6)	20.7 (4.5)
B	Both	3.7 (2.3)	10.3 (3.7)	7.6 (2.5)	11.7 (3.9)
	Control	26.5 (7.2)	11.2 (4.5)	9.3 (3.1)	21.2 (5.6)^*^
	Eyes	12.7 (3.5)	15.4 (4.0)	16.4 (3.6)	27.3 (5.3)
	Smell	8.7 (3.0)	9.6 (2.9)	8.6 (2.3)	7.4 (2.1)^*^
C	Both	16.4 (7.6)	10.5 (4.0)	5.7 (1.9)	7.7 (2.6)^∧^
	Control	12.0 (8.7)	7.5 (4.1)	6.1 (2.4)	10.1 (3.5)
	Eyes	3.8 (2.8)	8.6 (3.2)	6.0 (1.9)	10.1 (3.0)
	Smell	22.4 (10.2)	8.3 (4.6)	5.4 (2.6)	25.5 (6.3)^∧^
D	Both	4.7 (2.9)	3.8 (2.1)	2.5 (1.2)	3.1 (1.3)
	Control	2.9 (1.8)	1.6 (1.1)	2.3 (1.2)	1.9 (1.0)
	Eyes	3.9 (2.9)	3.4 (2.5)	2.8 (1.9)	1.3 (1.0)
	Smell	2.3 (2.3)	1.6 (1.6)	2.8 (1.8)	3.5 (2.0)

* *Significant difference between “control” and single prime condition (p = 0.03)*.

∧ *Significant difference between “both” and single prime conditions (p = 0.02)*.

In summary, the three hypotheses set out in the introduction were rejected. Regarding objective 1, the primes did not consistently increase the percentage of people who used the gel or soap dispensers. Of interest is the significant increase in soap use on Ward A during the both condition. The fact that a significant increase occurred here, but not for other wards and not for gel use, may suggest that contextual variables moderate the effectiveness of these priming conditions. Regarding objective 2, the amount of material used at the gel and soap dispensers also did not differ across conditions. And lastly, regarding objective 3, the effects of the primes did not differ by gender or hospital role.

##### Follow Up Cross Sectional Survey

To understand why the primes investigated in the main study did not increase hand hygiene compliance in the manner expected, we conducted a follow up study. For this purpose, we surveyed hospital staff, who were employed in the same hospital as the main study participants.

The particular follow-up survey instrument we employed was developed and validated by Dyson et al. (22). It measures attitudes and behaviors pertaining to health-workers' hand hygiene, predicated on the Theoretical Domains Framework (23). The purpose of this framework and the self-report instrument accompanying it is to enable interventionists to understand and deploy a variety of psychological techniques to support behavior change. The initial variant the framework encompassed 12 domains: “Knowledge,” “Skills,” “Social/professional role and identity,” “Beliefs about capabilities,” “Beliefs about consequences,” “Motivation and goals,” “Memory attention and decision processes,” “Environmental context and resources,” “Social influences,” “Emotion regulation,” “Behavioral regulation,” and “Nature of the behavior.” Dyson et al.'s survey instrument incorporates items pertaining to 10 of these domains. The number of domains was reduced as the results of their confirmatory factor analysis that led them to combine two of the original domains into a single domain called “Knowledge and Skills.” In addition, they did not include the original “Nature of behavior” domain because they determined that it was not a determinant of whether the desired behavior was enacted, but rather a description of the behavior itself.

To better understand the results of the present trial, the domains of most interest were “Memory and attention” and “Social influences.” “Memory and attention” was of interest, because, as outlined earlier, the primes of interest should have made the behaviors we wanted to influence more accessible in our main study participants' memory, and in so doing increased the likelihood that those behaviors would have actually occurred (12, 13). If “Memory and attention” was not a barrier to hand hygiene compliance in the hospital at the focus of the present research, then it is unlikely our olfactory primes could have promoted the behavior of key concern. “Social influences” were of interest because the visual prime should have evoked memories of social norms that encourage hand hygiene promoting behavior. If social norms prevailing in the focal hospital did not already encourage adherence to hand hygiene protocols *in situ*, it is unlikely that our visual prime would have triggered the desired behaviors. The “Memory and attention” and “Social influences” domains of primary interest in this follow up survey are incorporated in both the original and subsequently validated variant of the Theoretical Domains Framework (23, 24). The additional 8 domains highlight potential barriers and enablers which our priming interventions did not target. Nevertheless, we included them in this follow up survey for the sake of completeness.

## Methods

The Health Research Authority approved the follow-up survey as an amendment to the initial trial. In total 520 surveys were distributed between May and July 2018. To distribute the surveys a researcher asked ward managers to circulate copies of the instrument to all the staff whom they expected to clean their hands before interacting with patients. Each copy of the self-report questionnaire was placed in an envelope, along with a return envelope and an information sheet. A small piece of chocolate was attached to the envelope as a thank you for participants' time. In line with the ethical approval, and as stated in the participant information sheet, participants' consent to be part of the survey research was assumed if they returned the questionnaire. The questionnaire commenced by asking participants to state their gender and job title. Then it asked them to rate their own hand hygiene behaviors in response to a series of 35 items, using a 7-point Likert scale, where 1 represented “strongly agree” and 7 represented “strongly disagree.” The items were scored such that higher scores indicate greater barriers. For example, an item designed to capture “Memory and attention” read “Sometimes I miss out hand hygiene simply because I forget it” (reverse coded), and an item designed to capture “Social Influences” read “My hand hygiene is encouraged by others.” The data contained in the returned surveys were transferred to an Excel file and each item's median score along with its 25^th^ quartile and 75^th^ quartile responses were computed.

## Results

Of the 520 distributed surveys, 97 were returned (representing an 18.7% response rate). Seventy-nine of the participants identified as female, 14 as male, and 4 did not say. Regarding their role at hospital, 8 participants identified as doctors, 42 as nurses, 46 as other staff, and 1 did not say.

Table 6 shows participants' 50th (median), 25^th^ and 75th percentile responses to each item (75–25^th^ percentile gives the interquartile range). The items are arranged in order from the lowest to highest barrier. For “Memory and attention” participants noted low barriers for all three items (Medians = 1, 1, and 2). For “Social influences” participants noted higher barriers for three of the four items (Medians = 2, 4, 4, and 5).

**Table 6:** Median responses to each item surrounded by its interquartile range.

**Domain**	**Item**	**Number**	**Median**	**25th percentile**	**75th percentile**
Professional role	I engage in hand hygiene out of respect for my patients.	97	1.00	1.00	2.00
	Hand hygiene is a non-negotiable part of my role.	97	1.00	1.00	1.00
	Hand hygiene is part of my professional culture.	96	1.00	1.00	1.00
Knowledge and skills	Hand hygiene training is available to me.	97	2.00	1.00	4.00
	There are adverts or newsletters about hand hygiene in my workplace.	96	1.00	1.00	2.00
	Hand hygiene guidelines are easily accessible.	94	1.00	1.00	2.00
Memory and attention	(rs) Sometimes I miss out hand hygiene simply because I forget it.	97	1.00	1.00	3.50
	(rs) Hand hygiene is not second nature for me.	97	1.00	1.00	2.00
	(rs) I am more likely to forget hand hygiene if I am tired.	97	2.00	1.00	4.00
Motivation and goals	(rs) I feel complacent about hand hygiene.	92	2.00	1.00	4.75
	(rs) I cannot be bothered with hand hygiene.	97	1.00	1.00	1.00
	(rs) I disagree with some parts of the hand hygiene guidelines.	95	1.00	1.00	3.00
Beliefs about capability	(rs) There are some practical barriers to hand hygiene because of my particular job/role.	96	1.00	1.00	3.00
	(rs) I am reluctant to ask others to engage in hand hygiene.	94	2.00	1.00	5.00
	(rs) The frequency of hand hygiene required makes it difficult for me to carry it out as often as necessary.	94	2.00	1.00	4.00
Emotion	I am confident in my ability to carry out hand hygiene.	96	1.00	1.00	2.00
	I feel angry if hand hygiene is not carried out by others.	97	2.00	1.00	3.00
	I feel frustrated when others omit hand hygiene.	95	2.00	1.00	4.00
	I feel guilty if I omit hand hygiene.	93	2.00	1.00	3.00
	I feel ashamed if I omit hand hygiene.	94	1.00	1.00	4.00
Beliefs about consequences	If I do not engage in hand hygiene I may catch an infection.	97	1.00	1.00	1.50
	If I omitted hand hygiene I would blame myself for infections.	96	2.00	1.00	3.00
	If I engage in hand hygiene it improves patient confidence.	95	1.00	1.00	2.00
	If I miss out hand hygiene I will be subject to disciplinary action.	94	4.00	3.00	6.00
Action planning	Government targets have led to improvements in my hand hygiene.	97	3.00	1.00	5.00
	Hospital targets relating to infection or hand hygiene has led to improvements in my hand hygiene.	94	2.00	1.00	4.00
	Some strategies designed to improve hand hygiene influence my practice.	93	3.00	2.00	4.00
Environmental resources	(rs) It is difficult for me to attend hand hygiene courses due to time pressure.	96	4.00	2.25	5.75
	(rs) Some government targets make hand hygiene more difficult (Such as high bed occupancy).	93	3.00	1.00	5.00
	(rs) My environment is cluttered.	95	3.00	1.00	5.00
	(rs) My area of work has poor staffing levels.	94	4.00	2.00	6.00
Social influences	When staff engage in hand hygiene they are praised.	97	5.00	4.00	7.00
	I engage in hand hygiene because I do not want to let the team down.	96	2.00	1.00	5.00
	Supervision from senior staff means that carrying out hand hygiene is easier for me.	96	4.00	3.00	7.00
	My hand hygiene is encouraged by others.	96	4.00	2.00	6.00

*Items were reverse coded where applicable (indicated with “rs”) so that higher scores indicate greater barriers to hand hygiene*.

Regarding the remaining domains, participants noted higher barriers for the “Environmental resources” and “Action planning” domains. In terms of the former, staff experienced the following barriers to hand hygiene: time pressure (Median = 3), government targets (Median = 3), environmental clutter (Median = 3), and poor staffing (Median = 4). Regarding “Action planning” staff experienced the following as barriers: hospital targets (Median = 2), government targets (Median = 3), and other strategies (Median = 3).

## General Discussion

The current trial examined whether the benefits of priming interventions found to increase gel dispenser use in the United States (16, 17) generalized to the United Kingdom in a larger and longer randomized crossover trial. Our hypotheses for all three objectives were rejected. The primes did not consistently increase the percentage of people who used the gel or soap dispensers (Objective 1), or the amount of material used (Objective 2), and the effects of the primes did not differ by gender or hospital role (Objective 3).

With regards to the priming literature, this means that we did not obtain support for the generalizability of either of the primes investigated across countries or dispenser types. There was not any evidence to demonstrate that either of the two primes endured over the longer time periods encompassed by the present study, and there was not evidence that the effects of the primes were greater when presented in combination rather than in isolation. The significant increase in soap use on Ward A during the both condition may suggest that contextual variables moderate the effectiveness of these priming conditions. As a reminder, ward A specialized in renal medicine, while the other wards specialized in gastrointestinal surgery, hematology/oncology, and admissions. Further contextual variables were not captured in the current study, and a future study with deliberate intentions to assess such factors would be needed to assert this more confidently.

The follow-up survey revealed that the barriers to, and enablers of, hand hygiene experienced by staff employed at the hospital that was the focus of the present research were unlikely to be overcome with the olfactory and visual primes investigated. Specifically, staff did not believe “Memory and attention” was a large barrier to their hand hygiene, and therefore interventions that prime memory for hand hygiene may be unlikely to succeed. Further, staff did not believe existing “Social influences” positively influenced hand hygiene, and therefore interventions that prime existing social norms may be unlikely to succeed. The results also highlighted two other barriers that future interventions could target: “Environmental resources” and “Action planning”.

Regarding the “Environmental resources” barrier, other studies have successfully increased hand hygiene by increasing the salience of environmental resources already available; for example, by making the dispensers more salient with flashing lights (25) and auditory cues (26). Increasing the salience of the two primes investigated in the present trial, however, would have been difficult. For example, increasing the intensity of the olfactory prime would probably have resulted in more staff and visitor complaints, while increasing the size of the visual prime may well have occluded other posters the hospital deemed no less important.

The practical focus of the present trail may have enabled for a number of “real-world factors” to contribute background noise or error variance, thereby attenuating the effects of primary interest. For example, the current trial did not control for ward specific factors. One factor specific for wards A and B, but not C and D, was their history of infections that may have made people working within those wards more sensitive to our primes. However, as no social/physical factors were manipulated or systematically measured, this should be interpreted as a speculative point for future research. In addition, it is also an open question whether the effectiveness of the intervention would be different now, during the COVID-19 pandemic.

Another limitation of the present trial is the fact that we focused our data collection efforts within one hospital and solely at the ward entrances. We did so primarily to mirror as closely as possible the successful priming study of King et al. (17). Another potential limitation of the present study is that the focal hospital's policies required staff to use gel and soap when entering wards, and this is not one of the World Health Organization's recommended five moments for hand hygiene, which include (1) before touching a patient, (2) before clean/aseptic procedures, (3) after body fluid exposure/risk of exposure, (4) after touching a patient, and (5) after touching patients' surroundings (27). The fact that the current trial's primary outcome did not fall within international guidelines may help to explain why the hand hygiene rates we observed were so low.

A low rate of hand hygiene may itself signal a barrier to improvement. Put another way, it may be that a critical mass of people already cleaning their hands at ward entrances is necessary before nudges can facilitate the spread of that behavior. Simulations of linguistic behaviors suggest that this critical mass may be just 10% (28), while studies of online coordination suggest that the critical mass may be 25% (29). Even higher critical masses may be needed to overturn undesirable complex behaviors, as a previous study found a 40% mass needed to overturn undesirable gender conventions (30). The current research team posits that hand hygiene is a complex behavior that requires a relatively high critical mass to enable systemic change at the level required to ensure mass adherence to the protocols in place.

Alternatively, a study published after our trial suggests that the reminders should be of a social nature (31). This study surveyed 225 participants (staff, visitors, and patients) who did not use the gel dispenser upon entering the lobbies in various healthcare settings. When approached, researchers reminded participants to use the gel dispenser and asked participants whether it was important for people to clean their hands when entering hospital. No one refused to use the gel dispenser, and over 90% agreed it was important to do so. Plausibly, non-social reminders (e.g., posters) are not sufficient to build the habits people need to actualize their good intentions and so more social reminder may be necessary. If social reminders are used, a variable interval schedule of reinforcement is probably the most effective way to condition people's hand hygiene habits, another interesting idea that needs to be investigated in future research (32).

## Conclusions

In conclusion, the current trial was conducted to test the effectiveness of priming interventions to increase hand hygiene at ward entrances. Neither of the primes, in isolation or combination, yielded consistent benefits. As hand hygiene is an important component of all hospitals' infection prevention and control strategies, and current hand hygiene rates remain lower than desired, further evaluations of novel interventions should be encouraged. To increase the chance that such interventions are successful, research teams should initially assess the barriers to and enablers of hand hygiene that people in the relevant setting experience before attempting to apply remedies found to be successful in other populations and domains of application removed from the immediate context of healthcare settings (33).

## Data Availability Statement

The datasets presented in this article are not readily available because any request is subject to the permission of University Hospitals Birmingham NHS Foundation Trust for researchers who meet the criteria for access to confidential data. The original data that support the findings in this article will be made available from the lead author (Kelly Ann Schmidtke) or chief investigator (Ivo Vlaev) upon reasonable request, subject to the permission of University Hospitals Birmingham NHS Foundation Trust for researchers who meet the criteria for access to confidential data. Requests to access the datasets should be directed to Kelly.A.Schmidtke@warwick.ac.uk.

## Ethics Statement

The studies involving human participants were reviewed and approved by Health Research Authority (16/SC/0554). Written informed consent from the participants' legal guardian/next of kin was not required to participate in this study in accordance with the national legislation and the institutional requirements.

## Author Contributions

The trial and survey were collaboratively developed and composed by all authors, who made substantial contributions to the conception, design of the work, interpretations the data, and the write up of the manuscript: KS, NA, TM, AH, GH, KA, DB, and IV. KA was responsible for running trial analyses. KS was responsible for running survey analyses. LK and GH made major revisions to a redrafted manuscript. All authors have approved the submitted version and have agreed to be personally accountable for their own contributions to ensure that questions related to the accuracy and integrity of the work are appropriately investigated, resolved, and documented in the literature.

## Funding

This work was funded by the Health Foundation's Behavioral Insights Research Programme (Grant no. 7601) in a grant obtained by IV, TM, DB, AH, GH, and KS. The Health Foundation is an independent charity committed to bringing about better health and health care for people in the United Kingdom. Write up and further analyses for this project were supported by the National Institute for Health Research (NIHR) Applied Research Centre (ARC) West Midlands (NIHR200165) in an institution grant on which KS was employed during the write up.

## Author Disclaimer

The views expressed are those of the author(s) and not necessarily those of the NIHR, ARC, or the Department of Health and Social Care.

## Conflict of Interest

The authors declare that the research was conducted in the absence of any commercial or financial relationships that could be construed as a potential conflict of interest.

## Publisher's Note

All claims expressed in this article are solely those of the authors and do not necessarily represent those of their affiliated organizations, or those of the publisher, the editors and the reviewers. Any product that may be evaluated in this article, or claim that may be made by its manufacturer, is not guaranteed or endorsed by the publisher.
