# Supplementary material for: A Crossover Randomized Controlled Trial of Priming Interventions to Increase Hand Hygiene at Ward Entrances
Source: Front Public Health. 2022 Jan 17;9:781359. doi: 10.3389/fpubh.2021.781359 (PMC8801705; doi:10.3389/fpubh.2021.781359)
Supplement: Supplementary file 1 [file Data_Sheet_1.docx]

**Supplemental Materials 1.**

Analysis of the data according to the published protocol.

A generalized linear mixed model was used where the fixed effects were phase, ward, and the interaction of phase and ward, and a random intercept was included in the model to account for the repeated measurement over multiple weeks within each phase. The analysis results in Table S1 show that the interaction was highly significant. Therefore, subsequent analyses were conducted based on only phase 1 data. The Tukey-Kramer adjusted comparisons among the conditions resulted in the Both and Control conditions not being significantly different; however, these two conditions were significantly more effective than either the Eyes or Smell condition (Table S2).

Table S1. Analysis of the cluster randomized crossover trial for gel.

| Effect | F | P |
| --- | --- | --- |
| phase | 0.60 | 0.623 |
| ward | 42.70 | <0.001 |
| phase*ward | 5.05 | <0.001 |

n=9811

Table S2. Analysis with only phase 1 data for gel.

| Effect | F | P |  |  |  |  |
| --- | --- | --- | --- | --- | --- | --- |
| Condition | 11.06 | <0.001 |  |  |  |  |
|  |  |  |  |  |  |  |
|  |  |  |  | Comparison P-values | | |
| Condition | Mean | SE |  | Both | Control | Eyes |
| Both | 0.13 | 0.04 |  |  |  |  |
| Control | 0.16 | 0.05 |  | 0.626 |  |  |
| Eyes | 0.02 | 0.01 |  | <0.001 | <0.001 |  |
| Smell | 0.14 | 0.04 |  | 0.941 | 0.936 | <0.001 |

n=1528

The same generalized linear mixed model was used to understand use of the soap dispenser. The analysis results in Table S3 show that the interaction was not significant, but the effect of ward was still very significant. Table S4 presents the analysis results for just phase 1 data with Tukey-Kramer adjusted comparisons among the conditions. Again, the Both and Control conditions were not significantly different, but these two conditions were significantly more effective than either the Eyes or Smell condition.

Table S3. Analysis of the cluster randomized crossover trial for soap.

| Effect | F | P |
| --- | --- | --- |
| phase | 1.74 | 0.192 |
| ward | 26.02 | <0.001 |
| phase*ward | 1.38 | 0.191 |

n=9811

Table S4. Analysis with only phase 1 data for soap.

| Effect | F | P |  |  |  |  |
| --- | --- | --- | --- | --- | --- | --- |
| Condition | 11.06 | <0.001 |  |  |  |  |
|  |  |  |  |  |  |  |
|  |  |  |  | Comparison P-values | | |
| Condition | Mean | SE |  | Both | Control | Eyes |
| Both | 0.11 | 0.03 |  |  |  |  |
| Control | 0.08 | 0.02 |  | 0.481 |  |  |
| Eyes | 0.03 | 0.01 |  | 0.002 | <0.001 |  |
| Smell | 0.01 | 0.01 |  | 0.744 | 0.002 | 0.151 |

n=1528
